# Sprint velocity and step frequency during repeated sprint ability testing are associated with high-intensity locomotor activity in elite female field hockey

**DOI:** 10.1371/journal.pone.0351128

**Published:** 2026-06-08

**Authors:** Carlos Rivera, Pablo González-Frutos, Fernando Naclerio, Javier Mallo, Santiago Veiga

**Affiliations:** 1 Sports Department, Faculty of Physical Activity and Sport Sciences, Universidad Politécnica de Madrid, Madrid, Spain; 2 Real Sporting de Gijón, Asturias, Spain; 3 Faculty of Health Sciences, Universidad Francisco de Vitoria, Pozuelo de Alarcón, Madrid, Spain; 4 Institute for Lifecourse Development, School of Human Sciences, Centre for Exercise Activity and Rehabilitation, University of Greenwich, Eltham, United Kingdom; Murdoch University, AUSTRALIA

## Abstract

Field hockey is an intermittent, high-intensity team sport in which global positioning system (GPS) monitoring and field-based fitness testing are widely used; however, the relationship between repeated sprint ability (RSA) determinants and match physical demands remains unclear. This study examined the association between RSA kinematic variables (sprint velocity, step frequency, and step length) and GPS-derived locomotor activity metrics during elite female field hockey matches. Fourteen elite female field hockey players (all members of a first team competing at the highest national and European level) completed a linear RSA protocol consisting of six 30-m maximal sprints interspersed with 30 s of active recovery. External load was quantified during four official competitive matches played against top-level opponents (teams ranked in the top four of the national league or National Cup semi-finalists) within three weeks of testing using 10-Hz GPS devices. RSA sprint-velocity metrics showed very large correlations (r > 0.70, p < 0.01) with match performance variables as total sprint distance, sprint distance per minute (sprint·min ^-^¹), high-speed running per minute (HSR·min^-^¹) and high-intensity accelerations (>3 m·s^-2^·min ⁻ ¹). Step frequency also demonstrated very large associations (r > 0.70, p < 0.01) with sprint, sprint·min^-^¹, HSR·min^-^¹, and maximal acceleration, whereas step length was not significantly related to any match variable. These findings indicate that RSA performance, particularly sprint velocity and step frequency, is associated with high-intensity match demands in elite female field hockey. RSA testing may therefore represent a practical, low-cost tool for monitoring or approximating GPS-derived locomotor activity during competitive matches.

## Introduction

Field hockey is an intermittent team sport characterised by frequent high-intensity actions such as sprints, accelerations, decelerations and changes of direction [1]. These actions are considered key components of match play and have been widely analysed in relation to locomotor activity profiles [2–4]. In recent years, the use of Global Positioning System (GPS) devices has become widespread, enabling both the quantification and profiling of players’ locomotor activity during training and competition through metrics such as distance covered, speed, acceleration and high-intensity efforts [5,6].

Previous research has shown that physical demands in field hockey matches vary significantly depending on the level of competition, tactical strategies, and the quality of the opposition [1,6–9]. Generally, higher competitive levels are associated with increased high-intensity activity, reflected in elevated GPS variables such as high-speed running (HSR), sprinting, peak maximum speed, accelerations (Acc) >3 m·s^-2^ and decelerations (Dec) >3 m·s^-2^ [7,10]. However, even within elite competition, match-to-match variability is evident and is influenced by multiple contextual and situational factors, including competition format (e.g., league, tournament, or full-season analyses) [11–14,15], playing position (defenders, midfielders, and forwards) [14,16,15], match periods (Q1–Q4) [16,17], competitive context (e.g., national or international club competitions, or national teams) [12,16,15], sex comparisons [11], sport-specific characteristics [18], and environmental conditions (e.g., air temperature) [17].

In parallel with in-game monitoring, specific field tests are commonly used to assess fitness and readiness of the players [19–22]. Repeated Sprint Ability (RSA) tests are used to evaluate the capacity of athletes to perform repeated high-intensity efforts with minimal recovery between them [23]. Traditionally, the RSA has focused on total sprint time and fatigue indexes. Sprint performance is underpinned by mechanical and kinematic factors, with running velocity (V) determined by the interaction between step frequency (SF) and step length (SL) (V = SF × SL) Previous research has primarily examined the role of SF in the context of fatigue, demonstrating strong association between reduction in SF and increases in sprint time (r = 0.76–0.81, p < 0.01) [24]. Furthermore, fatigue-related changes in SF differ according to competitive levels (p < 0.05), with elite athletes exhibiting smaller decrements (2–5%) compared to sub-elite players (4–9%). [25]. Therefore, a better understanding of these kinematic responses may inform training strategies aimed to preserving SF and maintaining sprint performance under fatigue conditions.

Despite the widespread use of GPS tracking and RSA testing in elite sports environments, limited research has examined how the kinematic determinants of RSA performance relate to match-derived locomotor activity, particularly high-intensity running, sprinting, and acceleration-related variables. Understanding these relationships may enhance the practical relevance of RSA testing and support its use in locomotor activity monitoring in elite field hockey. Therefore, the primary aim of this study was to examine the associations between RSA kinematic variables (namely, sprint velocity, step frequency, and step length) and GPS-derived physical performance metrics (total distance, high-speed running, sprint distance, accelerations and decelerations, and maximum speed) during elite female field hockey matches. A secondary aim was to quantify match-to-match variation in RSA kinematic and GPS-derived performance measures across four monitored matches, in order to determine the stability of these variables and justify the use of averaged match data for correlational analyses.

It was hypothesized that RSA sprint velocity and step frequency would show stronger associations with high-intensity match locomotor activity variables (e.g., sprint distance, high-speed running, and accelerations) than step length. Additionally, it was hypothesized that the average values obtained across several matches between similarly ranked teams would demonstrate low match-to-match variability, thereby supporting their use as representative indicators of match locomotor activity.

## Materials and methods

### Participants

Twelve female field hockey players (age: 25.40 ± 4.48 years; height: 1.68 ± 0.04 m; body mass: 58.53 ± 2.56 kg), all of whom participated in all analysed matches, were included in this study. All players belonged to the same first team (first place in the Spanish league and second place in the Euro Hockey League). Additionally, eight players were members of the Spanish national team. According to the performance calibre framework proposed by McKay et al. [26], participants were classified as Tier 4 (elite level). Data collection was conducted between 2 December 2020 and 20 December 2020. The protocol was approved by the Local University Ethics Committee (Approval Code 35/2020), and all participants provided written informed consent in accordance with the Declaration of Helsinki after receiving a full explanation of the study procedures.

### Design

To ensure minimal disruption to the regular training program, coaching and support staff were informed of the study procedures and, where appropriate, involved in the study design, supervision, and test implementation. The RSA test was conducted during the physical conditioning phase of a mid-week training session and was consistently preceded by a standardized warm-up. The competitive matches analysed in the study occurred within three weeks of RSA testing, a timeframe considered sufficiently short to assume a relatively stable fitness status. These matches were played against teams ranked in the top four of the national league or National Cup semi-finalists, thereby representing a high competitive standard while reducing contextual variability between matches. In this context, the studied team scored fewer goals (2.5 vs. 4.8) and conceded more goals (0.75 vs. 0.60) than during the regular league season, further indicating the greater challenge posed by the analysed matches.

### RSA testing

A standardized warm-up which lasted 15 minutes preceded the RSA test, including 3 minutes of jogging and general movements, 2 minutes of dynamic stretching, 3 minutes of running drills, and 2 minutes of short accelerations over 5–10 m. The RSA protocol consisted of six maximal 30-m sprints, interspersed with 30 s of active recovery. During the recovery period, players decelerated for approximately 10 m and followed a triangular route to return slowly to the starting line. Each sprint began from a standing start, with the front foot placed 1 m behind the first timing gate. Maximal sprint intensity was verified by comparing peak sprint velocities obtained during the RSA test (20–30 m split) with match-derived peak speeds (27.1 ± 1.76 vs. 26.5 ± 1.7 km·h ⁻ ¹, respectively), and by analysing the performance decrement across repetitions (~1% in the second repetition and up to ~4% in the worst), consistent with previous findings in RSA protocols [24].

Sprint times were measured using electronic photocells (Microgate, Bolzano, Italy) positioned at 0 and 30 m, with the beam height adjusted to hip level of each player. Mean sprint velocity (V) was calculated as the ratio between distance covered (30 m) and sprint time (t). All sprints were recorded using a high-speed video camera (EX-ZR800; Casio Computer Co., Tokyo, Japan) placed laterally to the running lane operating at 60 Hz, 1/1000 s shutter speed, and 1920 × 1080-pixel resolution [25,27].

Video analysis enabled the determination of mean step frequency (SF), calculated by dividing the number of steps by the time to perform these steps, and mean step length (SL), calculated as the ratio between sprint velocity and step frequency. Although results are presented for the overall 0–30 m distance, step analysis was performed across three 10 m sections (0–10, 10–20, and 20–30 m), with an average of 6, 6, and 5 steps per section, respectively (≈17 steps in total). All analyses were conducted manually by a single experienced researcher (>15 years of experience in motion analysis). To assess the reliability of the procedure, repeated measurements (n = 30, non-consecutive trials) were performed, resulting in a root mean square error of 0.01 s for the total step time, corresponding to a variation of 0.07 km·h ⁻ ¹ in velocity and negligible differences in step length, indicating high reliability of the measurement procedure.

For each variable (sprint velocity, step frequency, and step length), five values were derived from the six repetitions: best, mean, worst, and two fatigue indexes (FI). These indexes were expressed as the percentage decrement in relation to the best sprint and calculated using both the mean (FImean = 100 – (mean/best*100)) and worst sprint performance (FIworst = 100 – (worst/best*100)) [25–28].

### GPS data

Data were collected using 10-Hz WIMU PRO GPS units (Realtrack Systems S.L., Almería, Spain; now part of Hudl, Lincoln, NE, USA), equipped with four triaxial accelerometers (1000 Hz) and three triaxial gyroscopes (1000 Hz), with a full-scale output range of 2000°·s^-^¹. The players wore the units in a pocket located on their upper back, between the shoulder blades, following the recommendations of the manufacturer to minimize measurement errors. These devices have been previously validated and shown to be highly reliable for monitoring performance in team sports [28,29]. Data analysis was performed using the SPRO software (Realtrack Systems S.L., Almería, Spain).

The following GPS-derived external load variables were analysed: total distance (TD; m), average speed (Avg speed; m·min^-^¹), high-speed running distance (HSR; 15–20 km·h ^-^¹; m), high-speed running per minute (HSR·min^-^¹; m·min^-^¹), sprint distance (Sprint; > 20 km·h^-^¹; m), sprint distance per minute (Sprint·min^-^¹; m·min^-^¹) [9,13,14,16], peak maximum speed (SP_max; km·h^-^¹), number of accelerations (Acc) and decelerations (Dec), accelerations and decelerations per minute (Acc·min^-^¹, Dec·min^-^¹; n·min^-^¹), peak maximum acceleration and deceleration (MaxAcc, MaxDec; m·s^-2^), number of accelerations and decelerations above 3 m·s^-2^ (Acc > 3, Dec > 3), and accelerations and decelerations above 3 m·s^-2^ per minute (Acc > 3·min^-^¹, Dec > 3·min^-^¹; n·min^-^¹).

Relative variables were calculated using total time on the field during matches, including all periods of play and stoppages (e.g., penalty corners, goals, substitutions, and other interruptions). In line with the primary aim of examining the associations between RSA kinematic variables (sprint velocity, step frequency, and step length) and match physical performance, sprint- and high-intensity-related metrics (HSR distance, HSR·min^-^¹, Sprint, Sprint·min^-^¹, SP_max, Acc > 3, Acc > 3·min^-^¹, and MaxAcc) were defined a priori as primary outcomes, as these variables represent the closest physiological and mechanical equivalents to RSA demands and were expected to show the strongest associations with RSA performance; in contrast, volume-based and general locomotor load variables (TD, Avg speed, Acc, Acc·min^-^¹, Dec, Dec·min^-^¹, Dec > 3, Dec > 3·min^-^¹, and MaxDec) were treated as secondary outcomes, providing contextual information on overall match demands and enabling the secondary aim of assessing match-to-match variability and the stability of averaged performance indicators.

### Statistical analysis

The statistical analyses were conducted using IBM Statistical Package for Social Sciences Statistics, version 29.0 (IBM Inc., Armonk, NY, USA) with alpha level set at p < 0.05. A repeated measures analysis of variance (ANOVA) was employed to compare four matches and the corresponding average values. Post hoc tests were conducted using Bonferroni corrections, and effect sizes *(ηp^2^)* were employed to estimate the magnitude of difference with 0.2, 0.5 and 0.8 indicating small, moderate or large effect sizes, respectively [29]. Prior to correlation analyses, normality was assessed using the Shapiro–Wilk test, and potential outliers were examined using z-scores. Furthermore, to explore the relationship between RSA values and the average match values, Pearson correlation coefficients were utilized with 0.1, 0.3, 0.5, 0.7 and 0.9 as thresholds for small, moderate, large, very large or nearly perfect correlations [30]*.*

## Results

### Match performance analysis

A significant main effects of match was observed for seven of the seventeen performance variables analysed: TD (F₂.₁₇ = 10.11, p < 0.001, η² = 0.48), Avg speed (F₁.₉₈ = 11.24, p < 0.001, η² = 0.51), Acc (F₂.₂₂ = 3.95, p = 0.029, η² = 0.29), Acc·min ⁻ ¹ (F₂.₅₅ = 9.11, p < 0.001, η² = 0.45), Dec (F₂.₂₂ = 4.01, p = 0.028, η² = 0.27), Dec·min ⁻ ¹ (F₂.₆₃ = 9.10, p < 0.001, η² = 0.45), and Dec3 (F₃.₀₀ = 7.23, p < 0.001, η² = 0.40). However, pairwise comparisons between the average match values (MatchAvg) and the individual matches revealed differences (p < 0.05) in five of the seventeen variables analyzed (Table 1). Specifically, Match 1 showed higher values for TD and Avg speed and lower values for Acc·min^-^¹ and Dec·min^-^¹ compared with MatchAvg. In contrast, Match 4 presented lower values for TD and Dec > 3·min^-^¹ compared with MatchAvg (Table 1).

**Table 1:** Comparison of GPS variables between individual matches and average match values.

	Match 1	Match 2	Match 3	Match 4	MatchAvg
**Primary outcomes**					
**HSR** **(m)**	896 (498)	912 (361)	883 (308)	715 (297)	852 (342)
**HSR·min ⁻ ¹** **(m·min ⁻ ¹)**	14.0 (6.7)	15.2 (4.1)	15.7 (4.6)	15.2 (4.3)	15.0 (4.6)
**Sprint** **(m)**	171 (161)	192 (120)	210 (107)	180 (90)	188 (102)
**Sprint·min ⁻ ¹** **(m·min ⁻ ¹)**	2.6 (2.4)	3.2 (1.7)	3.8 (1.9)	4.0 (2.1)	3.4 (1.5)
**SP_Max** **(km·h**^**-1**^)	24.1 (2.8)	25.0 (2.1)	25.2 (2.2)	24.7 (2.0)	24.8 (2.0)
**Acc > 3** **(n)**	16 (10)	17 (8)	14 (5)	13 (7)	15 (7)
**Acc > 3·min ⁻ ¹** **(n·min ⁻ ¹)**	0.26 (0.11)	0.28 (0.10)	0.27 (0.11)	0.28 (0.13)	0.27 (0.11)
**MaxAcc** **(m·s**^**2**^)	4.55 (0.69)	4.38 (0.52)	4.40 (0.37)	4.22 (0.70)	4.39 (0.37)
**Secondary outcomes**					
**TD** **(m)**	5838 (1972)	5153 (1730)	4708 (1299)	3969 (1111)	4917 (1379) ^*14^
**Avg speed** **(m·min ⁻ ¹)**	100.6 (12.4)	85.1 (10.0)	83.7 (11.9)	86.4 (8.3)	89.0 (8.1) ^*1^
**Acc** **(n)**	1861 (661)	1912 (556)	1814 (305)	1511 (382)	1774 (415)
**Acc·min ⁻ ¹** **(n·min ⁻ ¹)**	30.6 (2.2)	32.2 (2.3)	32.7 (1.5)	33.2 (1.3)	32.2 (1.5) ^*1^
**Dec** **(n)**	1861 (662)	1912 (557)	1814 (308)	1510 (382)	1774 (417)
**Dec·min ⁻ ¹** **(n·min ⁻ ¹)**	30.6 (2.2)	32.2 (2.3)	32.7 (1.6)	33.1 (1.3)	32.2 (1.5) ^*1^
**Dec > 3** **(n)**	33 (17)	32 (11)	28 (13)	22 (11)	29 (13)
**Dec > 3·min ⁻ ¹** **(n·min ⁻ ¹)**	0.51 (0.20)	0.54 (0.10)	0.50 (0.24)	0.47 (0.19)	0.50 (0.17) ^*4^
**MaxDec** **(m·s**^**2**^)	−5.40 (1.13)	−5.39 (0.62)	−5.41 (0.71)	−5.19 (0.74)	−5.35 (0.64)

*High speed running (HSR), high speed running per minute (HSR·min ⁻ ¹), sprint distance, sprint distance per minute (Sprint, Sprint·min ⁻ ¹), peak maximum speed in matches (SP_Max), ⁻ ¹), number of accelerations above 3m·s*^*2*^ *(Acc > 3), number of accelerations above 3m·s*^*2*^ *per minute (Acc > 3·min ⁻ ¹), peak maximum acceleration (MaxAcc), total distance absolute (TD), Avg speed (average speed), number of accelerations and decelerations (Acc, Dec) number of accelerations and decelerations per minute (Acc·min ⁻ ¹, Dec·min ⁻ ¹), number of decelerations above 3m·s*^*2*^ *(Dec > 3), number of decelerations obove 3m·s*^*2*^ per minute (Dec > 3·min ⁻ ¹) and peak maximum deceleration (MaxDec). Values are presented as mean (standard deviation). (*) Different from individual match at p < 0.05.

### Relationships between RSA Velocity (V) and match performance

Average match demands were positively associated with RSA velocity variables. Very large (p < 0.01) relationships were observed between V_Best (23.48 ± 0.97 km/h) and Sprint/min, and between V_Mean (22.9 ± 0.99 km/h) and V_Worst (22.5 ± 0.99 km/h) with HSR·min ⁻ ¹, Sprint, Sprint·min ⁻ ¹ and Acc > 3·min^-^¹ (Table 2). In addition, V_best showed large (p < 0.05) relationships with HSR·min^-^¹, Sprint, SP_Max and Acc3 > 3·min^-^¹. Likewise, V_mean and V_Worst showed large (p < 0.05) relationships with Avg speed, HSR, SP_Max, and Acc > 3. In contrast, FI_V_worst (4.14 ± 1.51%) showed large (p < 0.05) negative relationship with TD, while FI_V_mean (2.45 ± 1.02%) showed no significant relationship with any match variables.

**Table 2:** Relationships between RSA Velocity (best, mean and worst), index (mean and worst) and match performance (GPS variables).

	V_Best	V_Mean	V_Worst	FI_V_mean	FI_V_worst
**Primary outcomes**					
**HSR** **(m)**	0.50 (−0.11, 0.83)	0.60* (0.04, 0.87)	0.66* (0.13, 0.89)	−0.52 (−0.84, 0.08)	−0.51 (−0.84, 0.08)
**HSR·min ⁻ ¹** **(m·min ⁻ ¹)**	0.65* (0.13, 0.89)	0.76** (0.33, 0.93)	0.79** (0.39, 0.94)	−0.55 (−0.85, 0.04)	−0.47 (−0.82, 0.15)
**Sprint** **(m)**	0.64* (0.10, 0.89)	0.71** (0.22, 0.91)	0.74** (0.30, 0.92)	−0.39 (−0.79, 0.24)	−0.39 (−0.79, 0.24)
**Sprint·min ⁻ ¹** **(m·min ⁻ ¹)**	0.72** (0.27, 0.91)	0.78** (0.37, 0.94)	0.80** (0.41, 0.95)	−0.39 (−0.79, 0.24)	−0.34 (−0.76, 0.29)
**SP_Max** **(km·h**^**-1**^)	0.63* (0.08, 0.88)	0.69* (0.19, 0.91)	0.69* (0.19, 0.91)	−0.39 (−0.79, 0.24)	−0.25 (−0.71, 0.43)
**Acc > 3** **(n)**	0.51 (−0.09, 0.84)	0.61* ( 0.05, 0.88)	0.63* (0.08, 0.88)	−0.49 (−0.83, 0.11)	−0.40 (−0.79, 0.22)
**Acc > 3·min ⁻ ¹** **(n·min ⁻ ¹)**	0.64* (0.10, 0.89)	0.71** (0.22, 0.91)	0.70** (0.21, 0.91)	−0.41 (−0.80, 0.20)	−0.24 (−0.70, 0.44)
**MaxAcc** **(m·s**^**2**^)	0.51 (−0.09, 0.84)	0.58* (0.01, 0.87)	0.61* (0.05, 0.88)	−0.35 (−0.77, 0.28)	−0.35 (−0.77, 0.28)
**Secondary outcomes**					
**TD** **(m)**	0.07 (−0.53, 0.62)	0.19 (−0.43, 0.69)	0.27 (−0.36, 0.73)	−0.52 (−0.84, 0.08)	−0.57* (−0.86, 0.00)
**Avg speed** **(m·min ⁻ ¹)**	0.48 (−0.13, 0.83)	0.56* (−0.02, 0.86)	0.61* (0.05, 0.88)	−0.41 (−0.80, 0.20)	−0.42 (−0.80, 0.20)
**Acc** **(n)**	−0.13 (−0.66, 0.48)	−0.01 (−0.58, 0.57)	0.06 (−0.53, 0.61)	−0.47 (−0.82, 0.14)	−0.51 (−0.84, 0.09)
**Acc·min ⁻ ¹** **(n·min ⁻ ¹)**	−0.13 (−0.66, 0.48)	−0.09 (−0.63, 0.51)	−0.07 (−0.62, 0.52)	−0.10 (−0.64, 0.51)	−0.13 (−0.65, 0.48)
**Dec** **(n)**	−0.13 (−0.66, 0.48)	−0.01 (−0.58, 0.56)	−0.06 (−0.61, 0.52)	−0.47 (−0.82, 0.14)	−0.51 (−0.84, 0.09)
**Dec·min ⁻ ¹** **(n·min**^-^¹)	−0.13 (−0.65, 0.48)	−0.01 (−0.58, 0.57)	−0.07 (−0.62, 0.52)	−0.11 (−0.65, 0.49)	−0.14 (−0.66, 0.47)
**Dec > 3** **(n)**	0.19 (−0.43, 0.69)	0.30 (−0.33, 0.75)	0.37 (−0.26, 0.78)	−0.51 (−0.84, 0.09)	−0.52 (−0.84, 0.07)
**Dec > 3·min ⁻ ¹** **(n·min ⁻ ¹)**	0.34 (−0.35, 0.76)	0.44 (−0.23, 0.80)	0.47 (−0.20, 0.81)	−0.49 (−0.83, 0.11)	−0.43 (−0.80, 0.19)
**MaxDec** **(m·s**^**2**^)	−0.33 (−0.76, 0.30)	−0.42 (−0.80, 0.20)	−0.47 (−0.82, 0.14)	0.44 (−0.18, 0.81)	0.45 (−0.17, 0.81)

*High speed running (HSR), high speed running per minute (HSR·min ⁻ ¹), sprint distance, sprint distance per minute (Sprint, Sprint·min ⁻ ¹), peak maximum speed in matches (SP_Max), ⁻ ¹), number of accelerations above 3m·s*^*2*^ *(Acc > 3), number of accelerations above 3m·s*^*2*^ *per minute (Acc > 3·min ⁻ ¹), peak maximum acceleration (MaxAcc), total distance absolute (TD), Avg speed (average speed), number of accelerations and decelerations (Acc, Dec) number of accelerations and decelerations per minute (Acc·min ⁻ ¹, Dec·min ⁻ ¹), number of decelerations above 3m·s*^*2*^ *(Dec > 3), number of decelerations obove 3m·s*^*2*^ per minute (Dec > 3·min ⁻ ¹) and peak maximum deceleration (MaxDec). Values are presented as Pearson correlation coefficients (r), with 95% confidence intervals (CI) shown in parentheses. (*) Statistical correlation at p < 0.05. (**) Statistical correlation at p < 0.01.

### Relationships between RSA Step Length (SL) and match performance

No significant relationships were observed between average match demands and RSA step length variables (p > 0.05; Table 3).

**Table 3:** Relationships between RSA Step Length (best, mean and worst), index (mean and worst) and match performance (GPS variables).

	SL_Best	SL_Mean	SL_Worst	FI_SL_mean	FI_SL_worst
**Primary outcomes**					
**HSR** **(m)**	−0.39 (−0.79, 0.24)	−0.38 (−0.78, 0.25)	−0.42 (−0.80, 0.21)	0.22 (−0.40, 0.71)	0.35 (−0.29, 0.77)
**HSR·min ⁻ ¹** **(m·min ⁻ ¹)**	−0.28 (−0.74, 0.35)	−0.29 (−0.74, 0.34)	−0.35 (−0.77, 0.28)	0.36 (−0.27, 0.77)	0.47 (−0.14, 0.82)
**Sprint** **(m)**	−0.46 (−0.82, 0.15)	−0.45 (−0.81, 0.17)	−0.50 (−0.83, 0.11)	0.30 (−0.33, 0.75)	0.43 (−0.19, 0.80)
**Sprint·min ⁻ ¹** **(m·min ⁻ ¹)**	−0.41 (−0.80, 0.21)	−0.41 (−0.80, 0.21)	−0.47 (−0.82, 0.14)	0.40 (−0.26, 0.79)	0.52 (−0.09, 0.84)
**SP_Max** **(km·h**^**-1**^)	−0.28 (−0.74, 0.35)	−0.29 (−0.74, 0.34)	−0.35 (−0.77, 0.28)	0.36 (−0.27, 0.77)	0.47 (−0.14, 0.82)
**Acc > 3** **(n)**	−0.04 (−0.60, 0.55)	−0.04 (−0.60, 0.55)	−0.13 (−0.65, 0.48)	0.12 (−0.49, 0.65)	0.30 (−0.33, 0.75)
**Acc > 3·min ⁻ ¹** **(n·min ⁻ ¹)**	0.14 (−0.47, 0.66)	0.12 (−0.49, 0.65)	0.02 (−0.56, 0.57)	0.16 (−0.46, 0.67)	0.33 (−0.33, 0.76)
**MaxAcc** **(m·s**^**2**^)	−0.46 (−0.82, 0.15)	−0.47 (−0.82, 0.14)	−0.54 (−0.85, 0.05)	0.22 (−0.41, 0.70)	0.34 (−0.29, 0.76)
**Secondary outcomes**					
**TD** **(m)**	−0.37 (−0.78, 0.26)	−0.33 (−0.76, 0.30)	−0.32 (−0.75, 0.31)	0.14 (−0.47, 0.66)	0.22 (−0.40, 0.71)
**Avge speed** **(m·min ⁻ ¹)**	−0.06 (−0.61, 0.54)	−0.01 (−0.58, 0.56)	−0.02 (−0.59, 0.56)	0.04 (−0.54, 0.60)	0.15 (−0.46, 0.67)
**Acc** **(n)**	−0.36 (−0.77, 0.27)	−0.34 (−0.76, 0.29)	−0.32 (−0.76, 0.31)	0.06 (−0.53, 0.61)	0.12 (−0.49, 0.65)
**Acc·min ⁻ ¹** **(n·min ⁻ ¹)**	−0.08 (−0.63, 0.52)	−0.08 (−0.63, 0.52)	−0.08 (−0.63, 0.52)	−0.35 (−0.77, 0.28)	−0.32 (−0.76, 0.31)
**Dec** **(n)**	−0.36 (−0.77, 0.27)	−0.34 (−0.76, 0.29)	−0.32 (−0.76, 0.31)	0.06 (−0.54, 0.61)	0.12 (−0.49, 0.65)
**Dec·min ⁻ ¹** **(n·min ⁻ ¹)**	−0.09 (−0.63, 0.51)	−0.09 (−0.63, 0.51)	−0.09 (−0.63, 0.51)	−0.35 (−0.77, 0.28)	−0.32 (−0.75, 0.31)
**Dec > 3** **(n)**	−0.14 (−0.66, 0.47)	−0.13 (−0.65, 0.48)	−0.18 (−0.68, 0.44)	0.12 (−0.49, 0.65)	0.27 (−0.36, 0.73)
**Dec > 3·min ⁻ ¹** **(n·min ⁻ ¹)**	−0.01 (−0.58, 0.56)	0.02 (−0.56, 0.57)	−0.06 (−0.61, 0.53)	0.19 (−0.43, 0.69)	0.35 (−0.29, 0.77)
**MaxDec** **(m·s**^**2**^)	0.23 (−0.40, 0.71)	0.23 (−0.40, 0.71)	0.28 (−0.35, 0.74)	−0.06 (−0.61, 0.53)	−0.18 (−0.68, 0.44)

*High speed running (HSR), high speed running per minute (HSR·min ⁻ ¹), sprint distance, sprint distance per minute (Sprint, Sprint·min ⁻ ¹), peak maximum speed in matches (SP_Max), ⁻ ¹), number of accelerations above 3m·s*^*2*^ *(Acc > 3), number of accelerations above 3m·s*^*2*^ *per minute (Acc > 3·min ⁻ ¹), peak maximum acceleration (MaxAcc), total distance absolute (TD), Avg speed (average speed), number of accelerations and decelerations (Acc, Dec) number of accelerations and decelerations per minute (Acc·min ⁻ ¹, Dec·min ⁻ ¹), number of decelerations above 3m·s*^*2*^ *(Dec > 3), number of decelerations obove 3m·s*^*2*^ per minute (Dec > 3·min ⁻ ¹) and peak maximum deceleration (MaxDec). Values are presented as Pearson correlation coefficients (r), with 95% confidence intervals (CI) shown in parentheses. (*) Statistical correlation at p < 0.05. (**) Statistical correlation at p < 0.01.

### Relationships between RSA step frequency (SF) and match performance

Average match demands were positively associated with RSA step frequency variables. Very large (p < 0.01) relationships were observed between SF_Best (4.16 ± 0.28 Hz) and Sprint and Sprint·min^-^¹, and between SF_Mean (4.02 ± 0.28 Hz) and SF_Worst (3.94 ± 0.28 Hz) with HSR·min^-^¹, Sprint, Sprint·min ⁻ ¹ and MaxAcc (Table 4). In addition, SF_Best showed large (p < 0.05) relationships with HSR, HSR·min^-^¹, SP_max and MaxAcc. Likewise, SF_Mean and SF_Worst showed large (p < 0.05) relationships with HSR and SP_Max. In contrast, FI_SF_mean (1.62 ± 3.27%) showed large (p < 0.05) relationship with SP_Max, while FI_SF_worst (2.75 ± 5.16%) showed no significant relationship with any match variables.

**Table 4:** Relationships between RSA Step Frequency (best, mean and worst), index (mean and worst) and match performance (GPS variables).

	SF_Best	SF_Mean	SF_Worst	FI_SF_mean	FI_SF_worst
**Primary outcomes**					
**HSR** **(m)**	0.57* (−0.02, 0.86)	0.68* (0.16, 0.90)	0.68* (0.16, 0.90)	0.38 (−0.29, 0.78)	0.32 (−0.36, 0.74)
**HSR·min ⁻ ¹** **(m·min ⁻ ¹)**	0.60* (0.04, 0.87)	0.70** (0.21, 0.91)	0.71** (0.22, 0.91)	0.39 (−0.28, 0.79)	0.35 (−0.33, 0.76)
**Sprint** **(m)**	0.73** (0.27, 0.92)	0.80** (0.41, 0.95)	0.80** (0.41, 0.95)	0.47 (−0.20, 0.81)	0.43 (−0.24, 0.80)
**Sprint·min ⁻ ¹** **(m·min ⁻ ¹)**	0.75** (0.31, 0.93)	0.82** (0.46, 0.95)	0.81** (0.44, 0.95)	0.50 (−0.16, 0.83)	0.47 (−0.19, 0.81)
**SP_Max** **(km·h**^**-1**^)	0.63* (0.08, 0.88)	0.67* (0.14, 0.89)	0.65* (0.11, 0.88)	0.61* (0.05, 0.87)	0.54 (−0.06, 0.85)
**Acc > 3** **(n)**	0.37 (−0.30, 0.78)	0.41 (−0.26, 0.79)	0.38 (−0.29, 0.78)	0.32 (−0.36, 0.74)	0.24 (−0.44, 0.71)
**Acc > 3·min ⁻ ¹** **(n·min ⁻ ¹)**	0.33 (−0.35, 0.76)	0.34 (−0.34, 0.76)	0.31 (−0.37, 0.75)	0.25 (−0.43, 0.71)	0.21 (−0.47, 0.69)
**MaxAcc** **(m·s**^**2**^)	0.67* (0.14, 0.89)	0.74** (0.29, 0.92)	0.72** (0.26, 0.91)	0.35 (−0.33, 0.76)	0.29 (−0.39, 0.73)
**Secondary outcomes**					
**TD** **(m)**	0.28 (−0.39, 0.73)	0.38 (−0.29, 0.78)	0.39 (−0.28, 0.79)	0.46 (−0.22, 0.81)	0.39 (−0.28, 0.79)
**Avg speed** **(m·min ⁻ ¹)**	0.32 (−0.36, 0.74)	0.36 (−0.31, 0.77)	0.38 (−0.29, 0.78)	0.21 (−0.47, 0.69)	0.20 (−0.48, 0.68)
**Acc** **(n)**	0.15 (−0.52, 0.65)	0.26 (−0.42, 0.72)	0.26 (−0.42, 0.72)	0.35 (−0.33, 0.76)	0.26 (−0.42, 0.72)
**Acc·min ⁻ ¹** **(n·min ⁻ ¹)**	−0.08 (−0.62, 0.52)	0.00 (−0.57, 0.57)	−0.03 (−0.59, 0.55)	−0.31 (−0.75, 0.37)	−0.36 (−0.78, 0.31)
**Dec** **(n)**	0.15 (−0.52, 0.65)	0.26 (−0.42, 0.72)	0.26 (−0.42, 0.72)	0.35 (−0.33, 0.76)	0.26 (−0.42, 0.72)
**Dec·min ⁻ ¹** **(n·min ⁻ ¹)**	−0.08 (−0.62, 0.52)	0.00 (−0.57, 0.57)	−0.02 (−0.59, 0.55)	−0.30 (−0.75, 0.37)	−0.36 (−0.78, 0.31)
**Dec > 3** **(n)**	0.25 (−0.43, 0.71)	0.29 (−0.39, 0.73)	0.28 (−0.39, 0.73)	0.39 (−0.28, 0.79)	0.31 (−0.37, 0.75)
**Dec > 3·min ⁻ ¹** **(n·min ⁻ ¹)**	0.24 (−0.44, 0.71)	0.26 (−0.42, 0.72)	0.24 (−0.44, 0.71)	0.38 (−0.29, 0.78)	0.32 (−0.36, 0.74)
**MaxDec** **(m·s**^**2**^)	−0.34 (−0.76, 0.30)	−0.43 (−0.80, 0.20)	−0.42 (−0.80, 0.20)	−0.16 (−0.68, 0.55)	−0.10 (−0.64, 0.57)

*High speed running (HSR), high speed running per minute (HSR·min ⁻ ¹), sprint distance, sprint distance per minute (Sprint, Sprint·min ⁻ ¹), peak maximum speed in matches (SP_Max), ⁻ ¹), number of accelerations above 3m·s*^*2*^ *(Acc > 3), number of accelerations above 3m·s*^*2*^ *per minute (Acc > 3·min ⁻ ¹), peak maximum acceleration (MaxAcc), total distance absolute (TD), Avg speed (average speed), number of accelerations and decelerations (Acc, Dec) number of accelerations and decelerations per minute (Acc·min ⁻ ¹, Dec·min ⁻ ¹), number of decelerations above 3m·s*^*2*^ *(Dec > 3), number of decelerations obove 3m·s*^*2*^ per minute (Dec > 3·min ⁻ ¹) and peak maximum deceleration (MaxDec). Values are presented as Pearson correlation coefficients (r), with 95% confidence intervals (CI) shown in parentheses. (*) Statistical correlation at p < 0.05. (**) Statistical correlation at p < 0.01.

## Discussion

Firstly, in line with the secondary aim, the use of averaged match data across four games with similar ranking teams appears to be a valid and reliable approach, as only a limited number of GPS-derived variables (5 out of 17), mainly secondary volume-based metrics, showed significant match-to-match differences [7,31]. Although some between-match differences reached statistical significance, their practical magnitude should be interpreted in the context of the typical variability reported in larger datasets of international players [32]. This finding may support the use of aggregated data to reduce contextual noise and is in line with previous research advocating for averaged metrics during congested competitive periods [6]. Secondly, consistent with the primary aim and hypothesis, RSA sprint velocity demonstrated strong associations specifically with the predefined primary high-intensity match metrics (Sprint, Sprint·min ⁻ ¹, HSR·min ⁻ ¹, and Acc > 3·min ⁻ ¹), confirming that these variables may represent the closest mechanical and physiological equivalents to RSA demands [10,23], despite the RSA protocol being based on linear sprinting while match activity involves multidirectional and curved actions. Finally, RSA step frequency emerged as a more sensitive and consistent correlate of high-intensity performance than step length, further supporting the hypothesis that step frequency plays a more determinant role in linking RSA mechanical output to competitive match demands [24,25,33].

### Repeated measures GPS

Averaging physical performance data across several matches within a defined season or competition period is widely adopted in field hockey research [9,11,13,16,34,35]. In the current research, only five of the 17 parameters showed general and pairwise differences between MatchAvg and the four individual matches. This methodological approach aligns with previous findings during congested schedules, such as those reported by Romero-Moraleda [8], who observed similarities in Acc·min ⁻ ¹, Dec·min ⁻ ¹, and Dec > 3, along with variations in TD and Avg speed. In contrast, variables related to accelerations and decelerations —particularly at intensities above 3 m·s^2^ showed significant match-to-match variability, supporting earlier research that highlights their sensitivity to contextual and fatigue-related factors [8,36]. Notably, the fact that only five variables showed significant pairwise differences reinforce the notion/idea that certain GPS-derived metrics are more position-specific and context-dependent and, thus, tend to exhibit greater within-player and within-team variability [6,37,38].

The female field hockey players assessed in the present study demonstrated match running demands comparable to those reported for national-level competition in terms of TD and relative Avg speed [2,34,38]. However, values reported for the Spanish national team were higher for the TD (6856 m), Avg speed (137.3 m·min ⁻ ¹), and Sprint·min ⁻ ¹ (15.7 m·min ⁻ ¹), whereas HSR (>15 km·h ⁻ ¹; 845 m) and HSR·min ⁻ ¹ (16.8 m·min ⁻ ¹) were similar [16]. These discrepancies may be partly explained by differences in competition regulations between national and international play, such as quarter duration and the application of stopped time, as well as by reducing variability in playing standard with national teams compared with club competitions [16,18,38].

### Relationships between RSA velocity and match performance

The findings of the present study reinforce and extend the existing evidence provided by Rampinini et al. [31] in soccer regarding the construct validity of RSA as an indicator of match-related physical performance. While Rampinini et al. [31] reported significant correlations between RSA mean time and very high-intensity running (r = −0.60) and sprinting distance (r = −0.65), the current study revealed very large relationships between V_Best and Sprint·min ⁻ ¹, as well as moderate to large relationships between RSA metrics (V_Best, V_Mean, and V_Worst) and variables such as Avg speed, HSR·min ⁻ ¹, Acc3·min ⁻ ¹, and V_max. Additionally, the present study introduced the FI_V_Worst, showing a large negative association with total distance (TD), suggesting that greater fatigue, reflected in poorer performance in the worst sprint relative to the best, may be associated with a reduced capacity to sustain physical output throughout the match. This finding is consistent with the results reported by Rivera et al. [25], where female field-hockey players from a sub-elite team exhibited significantly higher fatigue indexes in sprint time than their elite counterparts. Together, these findings suggest that fatigue accumulation during repeated sprint efforts may be a relevant factor associated with performance level and the capacity to sustain match-related physical demands.

### Relationships between RSA step frequency and length and match performance

The findings of the present study align with previous research on the relevance of SF and SL in RSA performance. While average match demands and RSA outcomes in this study showed no significant associations with step length variables, very large and large relationships were observed between SF metrics (particularly SF_Best, SF_Mean, and SF_Worst) and key performance indicators such as sprint distance, sprint frequency, HSR·min ⁻ ¹, and maximal accelerations (Table 4). These results are in line with González-Frutos et al. [24], who also reported that fatigue-related changes in sprint time were correlated with SF fatigue indexes but not with SL, highlighting the importance of the control of frequency under fatigue constraints. Furthermore, although Rivera et al. [25] emphasized the importance of both SL and SF in RSA performance, SF showed decrements in later sprint segments (20–30 m) among lower-level players, consistent with the present findings where SF_Worst was associated with lower locomotor match outputs. Collectively, the observed results suggest that SF may represent a more sensitive and ecologically valid mechanical determinant than SL in explaining RSA performance and its relationship to competitive match demands.

### Limitations and practical applications

This study presents several limitations that should be acknowledged. First, the sample was limited to a single elite female team, and players were not grouped by playing position due to the inclusion criterion requiring participation in all analyzed matches, as well as variability in positional roles across games. The relatively small number of matches analysed (n = 4) should be considered when interpreting the findings, however, matches were selected within a short time frame and against similarly ranked opponents to reduce contextual variability. Second, although GPS systems may present constraints when capturing high-intensity accelerations and decelerations, the low match-to-match variability reported in high-level competition supports the use of averaged GPS-derived data for training prescription. The RSA protocol assessed linear sprint performance, which may not fully capture the multidirectional demands of match play. However, the observed associations suggest it remains a relevant underlying component. In addition, relative match variables were calculated using total time on the field, including stoppages, which may underestimate intensity compared to ball-in-play approaches. Furthermore, the relatively small sample size and the correlational design limit generalisability and preclude causal inference. Finaly, absolute speed thresholds were used to define high-speed running and sprint activity. Although commonly applied in field hockey to facilitate comparisons between studies [9,13, 14, 16], these thresholds may not represent the same relative intensity across players and should therefore be interpreted with caution [5].

From a practical perspective, the observed associations between RSA velocity metrics and match activity profiles highlight the relevance of integrating repeated-sprint and high-intensity running tasks into conditioning programs. Based on the mean values observed in the present study and previous literature [24,25], these metrics may be considered as potential reference values, suggesting that maintaining step frequency above 4 Hz during RSA testing and limiting velocity-related performance decrements to 4% between the best and worst sprint could be indicative of performance. Furthermore, the RSA test represents a low-cost and easy-to-administer assessment that may serve as a practical tool for talent identification and performance monitoring, providing an accessible alternative to GPS-based evaluations. Future research including larger samples and intervention-based designs is warranted to further refine these applications.

## Conclusions

External load variables showed limited match-to-match variability, supporting the use of averaged match data to characterize competitive demands. RSA sprint velocity metrics were strongly associated with high-intensity match activities, including sprinting, high-speed running, and accelerations, suggesting that RSA may represent a relevant indicator of high-intensity match locomotor activity. Furthermore, step frequency demonstrated stronger relationships with both RSA outcomes and match demands than step length, indicating greater sensitivity as a mechanical determinant of RSA performance. Collectively, our findings highlight the potential practical value of RSA testing, particularly sprint velocity and step frequency metrics, to monitor high-intensity performance capacity in elite female field hockey.

## Supporting information

S1 Dataset (XLSX)
