# Supplementary material for: Sprint velocity and step frequency during repeated sprint ability testing are associated with high-intensity locomotor activity in elite female field hockey
Source: PLoS One. 2026 Jun 8;21(6):e0351128. doi: 10.1371/journal.pone.0351128 (PMC13245795; doi:10.1371/journal.pone.0351128)
Supplement: Attachment — Submitted filename: Response to Reviewer 2.docx [file pone.0351128.s002.docx]

**Reviewer #2:**

Overall comments:
This manuscript examines the associations between repeated sprint ability (RSA) kinematic variables and locomotor activity in elite female field hockey players. The topic is relevant, and integrating RSA kinematics with GPS derived match metrics has the potential to contribute meaningfully to applied practice. The manuscript is generally well structured, and the rationale is clearly articulated. However, several important methodological and statistical details are missing, which limits confidence in the robustness of the findings.
The statistical analysis, in particular, requires greater methodological rigour. The manuscript does not justify the sample size or specify the variable on which the study was powered, and confidence intervals are not provided for key estimates. The reliance on simple correlations limits the ability to account for confounding factors or the repeated measures structure of the data. Mixed effects models or multivariable regression approaches could provide more robust insights by modelling within player variability and controlling for covariates. Addressing these issues would substantially strengthen the statistical validity and interpretability of the findings.

We would like to sincerely thank the reviewer for the thorough and insightful evaluation of our manuscript. We greatly appreciate the time and effort invested in providing such a detailed and constructive review. The comments have been extremely valuable in helping us improve the methodological clarity, statistical transparency, and overall interpretability of the study.

In response to these suggestions, we have carefully revised the manuscript, addressing all the points raised. Substantial improvements have been made, including clarification of methodological procedures, refinement of statistical reporting (e.g., inclusion of confidence intervals and assumptions checking), adjustments in terminology to ensure conceptual accuracy, and a more cautious interpretation of the findings in line with the correlational nature of the data. We have also incorporated additional references, expanded relevant sections of the Introduction and Methods, and explicitly acknowledged key limitations of the study. All changes are described in detail in the point-by-point responses provided below.

Title: Consider whether “match performance” is appropriate; the study examines locomotor activity, not performance outcomes.

We agree that the term “match performance” may be misleading, as the study specifically examines locomotor activity rather than direct performance outcomes. Accordingly, we have revised the title to better reflect the nature of the variables analyzed. We believe this change improves the accuracy and clarity of the manuscript.

Short title: Similarly, “match demands” is misleading. GPS/accelerometer data reflect activity profiles, not the true “demands” of match situations.

The short title has been revised to better reflect the nature of the variables analyzed.

Abstract: Replace “physical performance” with “GPS‑derived locomotor activity.”

Revised as suggested: ‘physical performance’ has been replaced with ‘GPS-derived locomotor activity’ in the Abstract.
L42: Fatigue development was not assessed; this claim should be removed.

The statement has been revised to remove any implication of fatigue assessment. The sentence now refers to the prediction of GPS-derived locomotor activity during competitive matches.

L49 – linking GPS to match outcomes is inappropriate. Greater locomotor activity may be a consequence of the standard of opponent and does not determine the ‘outcome’ (match result), as the sentence currently implies. See James et al. (2023). Relationships between opponent ranking and locomotor activity in international field hockey. International Journal of Sports Science & Coaching, 18(6), 2070-2081.
Feels like first paragraph is mostly redundant, given the number of preceding hockey GPS papers. I think the authors could probably start at Paragraph 2.

We agree that linking locomotor activity directly to match outcomes may be inappropriate, as match results are influenced by multiple contextual factors. Accordingly, the sentence has been revised to remove any implication of a direct relationship with match outcomes. In addition, the introductory paragraph has been streamlined to reduce redundancy and improve clarity.

L62 – 66. Suggest to attach the specific citation to each of these points about opponent / playing style / match importance. Environmental conditions are another modifying factor here, for example see James et al. (2022). Increased air temperature decreases high-speed, but not total distance, in international field hockey. Temperature, 9(4), 357–372.

We have revised the sentence to associate each contextual factor with specific references and have incorporated environmental conditions, including air temperature, as an additional factor influencing locomotor activity, as recommended.
L71 – 73 – the importance of kinematic factors is not explained. Authors may wish to expand on this. Why is it important to understand? Does it guide training prescription?

We thank the reviewer for this comment. We agree that the importance of kinematic factors was not sufficiently explained. The text has been revised to clarify their relevance by highlighting that sprint velocity is determined by the interaction between step length and step frequency (V = SL × SF), and that their analysis provides additional insight into the mechanisms of performance and fatigue, which may help to inform training prescription.

L78 – Clarify what is meant by “improving the ecological validity of RSA testing.” Are you implying RSA protocols should be modified based on these correlations?

The term ‘ecological validity’ has been replaced to better reflect the practical relevance of RSA testing in relation to GPS-derived locomotor activity.

L83 – ‘As a preliminary step’ not needed

The wording has been revised to remove ‘preliminary step’ and clarify the role of this analysis in supporting the use of averaged match data. For this reason, the results of the secondary aim are presented first in the Results section, as they provide the basis for the subsequent correlational analyses between match averages and RSA performance.

L85 – why were four matches selected?

The four matches were selected to ensure consistency with the RSA testing context and to include matches against top-ranked opponents, thereby reducing variability related to competitive level. This selection criterion is now clarified in the Methods section.

L85 – do you mean absolute total meters or intensity? (per min?). This is important to distinguish because you cannot control the playing time of the players.

As indicated in the Methods section, both absolute values and relative values normalized to individual playing time were included in the analysis.

L90 – what is similarly ranked?

The term ‘similarly ranked’ has been clarified. The analyzed team consistently ranked first or second in the national league over the past 10 years, and comparisons were made against teams occupying top positions in the league standings or reaching the semi-finals of the National Cup. This information has been clarified in the Methods section.

L91 – stability is inappropriate term for describing variability

The term ‘stability’ has been revised to ‘low match-to-match variability’ to more accurately describe the consistency of the measured variables.

L92 – this is not competitive performance, but locomotor activity.

The term ‘competitive performance’ has been replaced with ‘locomotor activity’ throughout the manuscript to ensure terminological accuracy.

L98 – Consider using the McKay et al. performance‑calibre framework to contextualise your players. doi:0.1123/ijspp.2021-0451

The performance calibre framework proposed by McKay et al. has been incorporated into the manuscript. Players were classified as Tier 4 (elite level), based on their participation in top-level national competitions, their performance as runners-up in the Euro Hockey League (the highest-level club competition in Europe), and the presence of several national team players.

L122 – 130 – Was maximal sprint speed (MSS) verified? How do you know players did not pace the first sprint? Can you compare RSA velocities with historical or match‑derived peak speeds?

Thank you for this important comment. Maximal sprint effort during the RSA test was verified using two complementary approaches. Although these analyses were initially performed to ensure the validity of the protocol, they were not included in detail in the manuscript in order to maintain clarity and focus on the main study objectives.

First, peak sprint velocity obtained during the RSA test (20–30 m split) was compared with match-derived peak speeds. RSA peak velocity (27.1 ± 1.76 km·h⁻¹) was slightly higher than match values (26.5 ± 1.7 km·h⁻¹; p = 0.02), indicating that players reached maximal or near-maximal sprinting speeds during the test.

Second, the evolution of performance across repetitions was analysed. A progressive decrease in sprint performance was observed (~1% in the second repetition and up to ~4% in the last), which is consistent with previously reported fatigue patterns in RSA protocols, suggesting that the initial sprint was performed maximally rather than paced.

These results support the notion that players did not adopt a pacing strategy and that the RSA test elicited maximal sprint efforts. In addition to the group-level decrement in performance, individual analysis confirmed that all players achieved higher sprint velocity in the first repetition compared to the second, and only three players exhibited slightly lower peak velocities (difference <1 km·h⁻¹) compared to match-derived values. Accordingly, a brief description of this verification has now been incorporated into the Methods section.

The description of step frequency and step length calculations is clear, but more detail is needed regarding the reliability of the video based method (e.g., intra and inter rater reliability, number of steps analysed, automated or manual digitisation).

Additional details regarding the video-based analysis have been incorporated into the Methods section.

Step frequency and step length were determined through manual video analysis across three 10 m sections (0–10, 10–20, and 20–30 m), with an average of approximately 17 steps analysed per sprint (≈6, 6, and 5 steps per section).

All analyses were performed by a single experienced researcher with more than 15 years of experience in motion analysis. To assess the reliability of the procedure, repeated measurements (n = 30, non-consecutive trials) were conducted, resulting in a root mean square error of 0.01 s for the total step time. This corresponds to minimal variation in derived velocity (0.07 km·h⁻¹) and negligible differences in step length, indicating high reliability of the measurement procedure.

L150 – please provide citation for use of predetermined speed thresholds. What are peak sprinting speeds for this group and does HSR actually represent high-speed for these individuals?

We have now added the corresponding references supporting their use in the Methods section. These predetermined thresholds have been previously used in female field hockey and team-sport literature, allowing comparisons across studies. We also acknowledge that absolute thresholds may not fully reflect the relative high-speed demands of each individual player. Therefore, this limitation has been explicitly addressed in the Limitations section, highlighting the variability in speed thresholds reported across studies. In this regard, a preliminary analysis using individualized thresholds was conducted, showing similar overall results. Accordingly, absolute thresholds were retained to facilitate comparison with previous literature. To provide additional context, peak maximum speed (SP_max) was included in the analysis and considered when interpreting high-speed and sprint activities.

Can you justify that 4 matches is sufficient beyond that you didn’t find a statistical difference between matches?

We agree that the absence of statistical differences alone does not justify the sufficiency of the number of matches analysed. In the present study, the selection of four matches was based on both practical and methodological considerations.

First, all matches were played within a short time frame (three weeks) against similarly ranked high-level opponents, which reduces contextual variability and supports the assumption of a relatively stable performance level. Second, previous research in elite team sports has reported relatively low match-to-match variability in GPS-derived external load variables, supporting the use of averaged values to characterise match activity profiles.

Additionally, our results showed that only a limited number of variables differed between individual matches and the average values, further suggesting a consistent locomotor profile across the analysed matches.

This aspect has been clarified in the manuscript, and the limited number of matches has been explicitly acknowledged as a study limitation.

Also, did all 14 players play all matches?

Thank you for this comment. We would like to clarify that an error was present in a previous version of the manuscript. The final analysis was conducted using only the 12 players who participated in all analysed matches, as indicated in the Participants section. This criterion was applied to ensure consistency across matches and avoid the influence of variations in playing time. The manuscript has been revised to ensure consistency in reporting this information.

Correlations are presented as “very large,” but the manuscript should include confidence intervals for correlation coefficients to report uncertainty. Additionally, the authors should clarify whether assumptions (normality, outliers) were checked.

We have now included 95% confidence intervals (CI) for all Pearson correlation coefficients in Tables 2–4 to better reflect the uncertainty of the estimates. In addition, the Statistical Analysis section has been updated to clarify that normality was assessed using the Shapiro–Wilk test and potential outliers were examined using z-scores. Furthermore, the wording in the Discussion and Conclusions has been slightly tempered to avoid overly strong interpretations and to better reflect the uncertainty and correlational nature of the findings.

Given the repeated measures structure (multiple matches nested within players), mixed effects models would provide a more statistically rigorous framework. Such models account for:
• Repeated measures,
• Incorporate relevant contextual covariates (e.g., position, playing time, opponent ranking), and
• estimate individual slopes linking RSA metrics to match performance.
Similarly, multivariable regression could help determine the independent contribution of sprint velocity, step frequency, and step length while controlling for shared variance. This would provide a more nuanced understanding of which RSA determinants truly predict match demands.

Thank you for this valuable suggestion. We agree that mixed-effects and multivariable models could provide a more comprehensive analysis. However, the study was based on a relatively small sample (n = 12) of players who participated in all matches, with some variability in positional roles across games. In this context, simpler correlation-based analyses were considered more appropriate to avoid overfitting and to facilitate interpretation for practitioners and coaches. This limitation has now been acknowledged in the manuscript. Future studies including larger samples and more stable contextual variables will aim to implement more advanced modelling approaches.

Table 1 – 2 decimal places is not necessary. Consider the reported accuracy of such systems when choosing the level of precision to report (e.g. 0 d.p. should be sufficient here for most variables aside of accel metrics).

We appreciate this suggestion. The number of decimal places has been adjusted according to the measurement accuracy of the GPS system. Specifically, absolute variables are now reported with no decimal places, while relative and acceleration-based variables retain one or two decimals where appropriate.

There is no such thing as TD.min or relative distance. This is an average speed. Average speed is typically expressed as m.min in training contexts and the literature.

We agree that the term m·min⁻¹ represents an average speed (i.e., distance covered per unit of time), and this terminology is commonly used in training contexts and the literature. However, in team sports research (particularly in field hockey and other intermittent sports) the term relative distance (m·min⁻¹) is also widely used to contextualize locomotor activity relative to effective playing time, especially when substitutions or unequal playing time are present. For example, previous studies have explicitly reported relative distance (m·min⁻¹) under this terminology [8]. Therefore, we believe that retaining the term relative distance is appropriate within this research context.

A lot of values in these tables. Did you consider a multi-panel plot showing match values and you could have a dashed line through the middle of each to represent the average?

We agree that multi-panel plots with reference lines can be a useful way to visualise trends across matches. However, given the number of variables included in the present study, we opted to retain the tabular format to allow readers direct access to the exact values for each match. This facilitates precise interpretation and practical application, particularly for practitioners who may wish to compare specific match outputs or contextualise individual performances. Therefore, we believe that the current tabular presentation provides greater clarity and usability for the intended audience, while still allowing straightforward interpretation of match-to-match variation.

L236 – “Average match demands and SL during the RSA test did not show any significant (p < 0.05) relationship (Table 3).” This p value is significant.

We apologize for the typo, as the p-value was incorrectly reported as significant. This has now been corrected in the revised manuscript.

Table 3 – commas instead of decimals throughout this table.

This has now been corrected throughout Table 3.

L280 – arguably the statistical significance is not the primary consideration here, probably more so the “clinical’ meaningfulness of those measures. See larger datasets for typical variability of international players e.g >70 matches. doi: 10.3389/fspor.2021.653364

We have revised the manuscript to better contextualize the magnitude of the observed match-to-match differences in relation to the typical variability reported in larger datasets of international players (e.g., James et al., 2021).

L292, suggest to discuss aim 1 first and then the variability analysis which was secondary.

We thank the reviewer for this suggestion. While the aims were initially ordered by importance, we structured the Results and Discussion sections to follow the analytical workflow of the study. Specifically, we first examined the consistency of the averaged match data, as this was necessary to support the subsequent analysis of the relationships with RSA performance. We believe this order improves the logical flow and facilitates interpretation for the reader.

L314 – no discussion of how data were processed – ball in play time only? What did you do for PCs and after goals scored. This artificially skews intensity metrics.

We thank the reviewer for this important comment. We acknowledge that the description of the data processing was not sufficiently detailed in the original version. We have now clarified in the Methods section that relative variables were calculated based on total time spent on the field, including all match periods and stoppages (e.g., penalty corners, goals, and substitutions). Additionally, we have acknowledged in the Limitations section that this approach may underestimate intensity-based metrics compared to ball-in-play methods.

The discussion would benefit from acknowledging that RSA testing captures linear sprinting, whereas match demands include curved runs and directional changes.

We agree that RSA testing reflects linear sprint performance, whereas match activity involves multidirectional and curved actions. This aspect has now been acknowledged as a limitation in the revised manuscript. However, the strong associations observed suggest that linear RSA performance remains a relevant underlying component of match activity in elite players.

The conclusion that RSA velocity and step frequency are “closely linked” to match performance should be tempered. Correlational data cannot establish predictive validity or causality.

We agree that the correlational nature of the data does not allow us to infer causality or predictive validity. Accordingly, the conclusion and abstract have been revised to adopt more cautious language, emphasizing associations rather than predictive or causal relationships.
