# Supplementary material for: Sprint velocity and step frequency during repeated sprint ability testing are associated with high-intensity locomotor activity in elite female field hockey
Source: PLoS One. 2026 Jun 8;21(6):e0351128. doi: 10.1371/journal.pone.0351128 (PMC13245795; doi:10.1371/journal.pone.0351128)
Supplement: Attachment — Submitted filename: Response_to_Reviewer_2_auresp_2.docx [file pone.0351128.s003.docx]

**Reviewer #2:**

Well done on making considerable changes. I think the manuscript is much better positioned bow, especially as many limitations are transparently reported.
The only minor comment i would make to the authors is to encourage them to again reconsider the use of 'relative distance' - just because other research has published this, is not a rationale to use scientifically incorrect terminology. Distance is not being normalized to some factor, it is simply divided by time, which provides speed.

Thank you for this insightful comment and for the positive evaluation of the revised manuscript. We agree that the term “relative distance” may be conceptually imprecise, as the variable reflects distance covered per unit of time and therefore represents an average speed measure rather than a normalized distance metric. Accordingly, the terminology has been revised throughout the manuscript by replacing “total distance per minute” and its abbreviation “TD·min⁻¹” with “average speed” and “Avg speed”, respectively, to improve conceptual and scientific accuracy.
