# Supplementary material for: Sprint velocity and step frequency during repeated sprint ability testing are associated with high-intensity locomotor activity in elite female field hockey
Source: PLoS One. 2026 Jun 8;21(6):e0351128. doi: 10.1371/journal.pone.0351128 (PMC13245795; doi:10.1371/journal.pone.0351128)
Supplement: Peer review history

# Peer review: Decision Letter 0

**DOI:** 10.1371/journal.pone.0351128.r001

**Authors:** Paul Goods

24 Mar 2026

PONE-D-26-08660

Sprint velocity and step frequency during repeated sprint ability testing are associated with high-intensity match performance in elite female field hockey

PLOS One

Dear Dr. González-Frutos,

Thank you for submitting your manuscript to PLOS ONE. After careful consideration, we feel that it has merit but does not fully meet PLOS ONE’s publication criteria as it currently stands. Therefore, we invite you to submit a revised version of the manuscript that addresses the points raised during the review process.

Please submit your revised manuscript by May 08 2026 11:59PM. If you will need more time than this to complete your revisions, please reply to this message or contact the journal office at plosone@plos.org. When you're ready to submit your revision, log on to https://www.editorialmanager.com/pone/ and select the 'Submissions Needing Revision' folder to locate your manuscript file.

Please include the following items when submitting your revised manuscript:

- A letter that responds to each point raised by the academic editor and reviewer(s). You should upload this letter as a separate file labeled 'Response to Reviewers'.
- A marked-up copy of your manuscript that highlights changes made to the original version. You should upload this as a separate file labeled 'Revised Manuscript with Track Changes'.
- An unmarked version of your revised paper without tracked changes. You should upload this as a separate file labeled 'Manuscript'.

If you would like to make changes to your financial disclosure, please include your updated statement in your cover letter. Guidelines for resubmitting your figure files are available below the reviewer comments at the end of this letter.

If applicable, we recommend that you deposit your laboratory protocols in protocols.io to enhance the reproducibility of your results. Protocols.io assigns your protocol its own identifier (DOI) so that it can be cited independently in the future. For instructions see: https://journals.plos.org/plosone/s/submission-guidelines#loc-laboratory-protocols. Additionally, PLOS ONE offers an option for publishing peer-reviewed Lab Protocol articles, which describe protocols hosted on protocols.io. Read more information on sharing protocols at https://plos.org/protocols?utm_medium=editorial-email&utm_source=authorletters&utm_campaign=protocols.

We look forward to receiving your revised manuscript.

Kind regards,

Paul SR Goods

Academic Editor

PLOS One

Journal requirements:

When submitting your revision, we need you to address these additional requirements.

1. Please ensure that your manuscript meets PLOS ONE's style requirements, including those for file naming. The PLOS ONE style templates can be found at

https://journals.plos.org/plosone/s/file?id=wjVg/PLOSOne_formatting_sample_main_body.pdf and

https://journals.plos.org/plosone/s/file?id=ba62/PLOSOne_formatting_sample_title_authors_affiliations.pdf

2. We note that you have indicated that there are restrictions to data sharing for this study. For studies involving human research participant data or other sensitive data, we encourage authors to share de-identified or anonymized data. However, when data cannot be publicly shared for ethical reasons, we allow authors to make their data sets available upon request. For information on unacceptable data access restrictions, please see http://journals.plos.org/plosone/s/data-availability#loc-unacceptable-data-access-restrictions.

Before we proceed with your manuscript, please address the following prompts:

a) If there are ethical or legal restrictions on sharing a de-identified data set, please explain them in detail (e.g., data contain potentially identifying or sensitive patient information, data are owned by a third-party organization, etc.) and who has imposed them (e.g., a Research Ethics Committee or Institutional Review Board, etc.). Please also provide contact information for a data access committee, ethics committee, or other institutional body to which data requests may be sent.

b) If there are no restrictions, please upload the minimal anonymized data set necessary to replicate your study findings to a stable, public repository and provide us with the relevant URLs, DOIs, or accession numbers. Please see http://www.bmj.com/content/340/bmj.c181.long for guidelines on how to de-identify and prepare clinical data for publication. For a list of recommended repositories, please see https://journals.plos.org/plosone/s/recommended-repositories. You also have the option of uploading the data as Supporting Information files, but we would recommend depositing data directly to a data repository if possible.

Please update your Data Availability statement in the submission form accordingly.

If the reviewer comments include a recommendation to cite specific previously published works, please review and evaluate these publications to determine whether they are relevant and should be cited. There is no requirement to cite these works unless the editor has indicated otherwise.

Additional Editor Comments:

Thank you for your submission. You will see that both reviewers are recommending substantial revisions to the manuscript in order for it to be considered for publication, and I'd invite you to carefully consider the points raised when preparing your response.

I look forward to receiving your revised manuscript.

[Note: HTML markup is below. Please do not edit.]

Reviewers' comments:

Reviewer's Responses to Questions

**Comments to the Author**

1. Is the manuscript technically sound, and do the data support the conclusions?

The manuscript must describe a technically sound piece of scientific research with data that supports the conclusions. Experiments must have been conducted rigorously, with appropriate controls, replication, and sample sizes. The conclusions must be drawn appropriately based on the data presented.

Reviewer #1: No

Reviewer #2: No

**********

2. Has the statistical analysis been performed appropriately and rigorously?

Reviewer #1: Yes

Reviewer #2: No

**********

3. Have the authors made all data underlying the findings in their manuscript fully available?

The PLOS Data policy requires authors to make all data underlying the findings described in their manuscript fully available without restriction, with rare exception (please refer to the Data Availability Statement in the manuscript PDF file). The data should be provided as part of the manuscript or its supporting information, or deposited to a public repository. For example, in addition to summary statistics, the data points behind means, medians and variance measures should be available. If there are restrictions on publicly sharing data—e.g. participant privacy or use of data from a third party—those must be specified.

Reviewer #1: No

Reviewer #2: Yes

**********

4. Is the manuscript presented in an intelligible fashion and written in standard English?

PLOS ONE does not copyedit accepted manuscripts, so the language in submitted articles must be clear, correct, and unambiguous. Any typographical or grammatical errors should be corrected at revision, so please note any specific errors here.

Reviewer #1: Yes

Reviewer #2: Yes

**********

5. Review Comments to the Author

Please use the space provided to explain your answers to the questions above. You may also include additional comments for the author, including concerns about dual publication, research ethics, or publication ethics. (Please upload your review as an attachment if it exceeds 20,000 characters)

Reviewer #1: Abstract:

Fourteen elite female field

30 hockey players (eight of them international-level standard)

- what level was the rest of the athletes?

"During four high-level competitive matches "

what does this mean? What is the level of competition (national? intra squad? international?

Intro:

Step frequency also

37 demonstrated very large associations with sprint, sprint·min⁻¹, HSR·min⁻¹, and maximal

38 acceleration

- please provide data to support "very large"

The relationship

73 between these factors and performance has been reported in the case of SF, and it may be

74 affected differently by fatigue and competitive level [22,23].

- this sentence could use more information on the relationships between factors - is it inverse? positive? how is it affected by fatigue and comp level?

GPS-derived physical performance metrics during elite

83 female field hockey matches.

- Which GPS metrics?

Materials and methods:

96 Participants

97 Fourteen elite female hockey players (age 25.40 ± 4.48 years, height 1.68 ± 0.04 m and

98 body weight 58.53 ± 2.56 kg), including eight international-level players participated in

99 this study.

- What was the level of the remaining players?

Were they analyzed or grouped by position? this would be an important factor to include in game data. This is a major limitation.

During these matches, the average number of

114 goals scored by the studied team was 2.5 compared with 4.8 during the regular league

115 season. Goals conceded averaged 0.75 against top-four teams and 0.60 against lower 116 ranked teams.

- This feels random here. Maybe more context needed? Is this indicating the context of the games played?

Were all players played in every game? was just active time on pitch included in analysis?

Table 1-4 - Horizontal lines between rows would improve readability

276 DISCUSSION

277 Firstly, in line with the secondary aim, the use of averaged match data across four games

278 with similar ranking teams proved to be a valid and reliable approach,

I think language coul dbe softened here, it does not necessarily prove antying, but is just 4 games so may suggest rather than prove

Practical applications like step frequency above 4hz and decrements below 4% feel like they are overstretching what the paper actually shows.

I think it is also important to acknowledge small sample size and use of correlational analyses in the limitaitons, and with any interpretations.

Reviewer #2: Overall comments:

This manuscript examines the associations between repeated sprint ability (RSA) kinematic variables and locomotor activity in elite female field hockey players. The topic is relevant, and integrating RSA kinematics with GPS derived match metrics has the potential to contribute meaningfully to applied practice. The manuscript is generally well structured, and the rationale is clearly articulated. However, several important methodological and statistical details are missing, which limits confidence in the robustness of the findings.

The statistical analysis, in particular, requires greater methodological rigour. The manuscript does not justify the sample size or specify the variable on which the study was powered, and confidence intervals are not provided for key estimates. The reliance on simple correlations limits the ability to account for confounding factors or the repeated measures structure of the data. Mixed effects models or multivariable regression approaches could provide more robust insights by modelling within player variability and controlling for covariates. Addressing these issues would substantially strengthen the statistical validity and interpretability of the findings.

Title: Consider whether “match performance” is appropriate; the study examines locomotor activity, not performance outcomes.

Short title: Similarly, “match demands” is misleading. GPS/accelerometer data reflect activity profiles, not the true “demands” of match situations.

Abstract: Replace “physical performance” with “GPS‑derived locomotor activity.”

L42: Fatigue development was not assessed; this claim should be removed.

L49 – linking GPS to match outcomes is inappropriate. Greater locomotor activity may be a consequence of the standard of opponent and does not determine the ‘outcome’ (match result), as the sentence currently implies. See James et al. (2023). Relationships between opponent ranking and locomotor activity in international field hockey. International Journal of Sports Science & Coaching, 18(6), 2070-2081.

Feels like first paragraph is mostly redundant, given the number of preceding hockey GPS papers. I think the authors could probably start at Paragraph 2.

L62 – 66. Suggest to attach the specific citation to each of these points about opponent / playing style / match importance. Environmental conditions are another modifying factor here, for example see James et al. (2022). Increased air temperature decreases high-speed, but not total distance, in international field hockey. Temperature, 9(4), 357–372.

L71 – 73 – the importance of kinematic factors is not explained. Authors may wish to expand on this. Why is it important to understand? Does it guide training prescription?

L78 – Clarify what is meant by “improving the ecological validity of RSA testing.” Are you implying RSA protocols should be modified based on these correlations?

L83 – ‘As a preliminary step’ not needed

L85 – why were four matches selected?

L85 – do you mean absolute total meters or intensity? (per min?). This is important to distinguish because you cannot control the playing time of the players.

L90 – what is similarly ranked?

L91 – stability is inappropriate term for describing variability

L92 – this is not competitive performance, but locomotor activity.

L98 – Consider using the McKay et al. performance‑calibre framework to contextualise your players. doi:0.1123/ijspp.2021-0451

L122 – 130 – Was maximal sprint speed (MSS) verified? How do you know players did not pace the first sprint? Can you compare RSA velocities with historical or match‑derived peak speeds?

The description of step frequency and step length calculations is clear, but more detail is needed regarding the reliability of the video based method (e.g., intra and inter rater reliability, number of steps analysed, automated or manual digitisation).

L150 – please provide citation for use of predetermined speed thresholds. What are peak sprinting speeds for this group and does HSR actually represent high-speed for these individuals?

Statistical approach - The manuscript does not provide any justification for the sample size (n = 14), nor does it specify which variable or effect the study was powered to detect.

Can you justify that 4 matches is sufficient beyond that you didn’t find a statistical difference between matches? Also, did all 14 players play all matches?

Correlations are presented as “very large,” but the manuscript should include confidence intervals for correlation coefficients to report uncertainty. Additionally, the authors should clarify whether assumptions (normality, outliers) were checked.

Given the repeated measures structure (multiple matches nested within players), mixed effects models would provide a more statistically rigorous framework. Such models account for:

• Repeated measures,

• Incorporate relevant contextual covariates (e.g., position, playing time, opponent ranking), and

• estimate individual slopes linking RSA metrics to match performance.

Similarly, multivariable regression could help determine the independent contribution of sprint velocity, step frequency, and step length while controlling for shared variance. This would provide a more nuanced understanding of which RSA determinants truly predict match demands.

Table 1 – 2 decimal places is not necessary. Consider the reported accuracy of such systems when choosing the level of precision to report (e.g. 0 d.p. should be sufficient here for most variables aside of accel metrics).

There is no such thing as TD.min or relative distance. This is an average speed. Average speed is typically expressed as m.min in training contexts and the literature.

A lot of values in these tables. Did you consider a multi-panel plot showing match values and you could have a dashed line through the middle of each to represent the average?

L236 – “Average match demands and SL during the RSA test did not show any significant (p < 0.05) relationship (Table 3).” This p value is significant.

Table 3 – commas instead of decimals throughout this table.

L280 – arguably the statistical significance is not the primary consideration here, probably more so the “clinical’ meaningfulness of those measures. See larger datasets for typical variability of international players e.g >70 matches. doi: 10.3389/fspor.2021.653364

L292, suggest to discuss aim 1 first and then the variability analysis which was secondary.

L314 – no discussion of how data were processed – ball in play time only? What did you do for PCs and after goals scored. This artificially skews intensity metrics.

The discussion would benefit from acknowledging that RSA testing captures linear sprinting, whereas match demands include curved runs and directional changes.

The conclusion that RSA velocity and step frequency are “closely linked” to match performance should be tempered. Correlational data cannot establish predictive validity or causality.

**********

6. PLOS authors have the option to publish the peer review history of their article (what does this mean?). If published, this will include your full peer review and any attached files.

If you choose “no”, your identity will remain anonymous but your review may still be made public.

**Do you want your identity to be public for this peer review?** For information about this choice, including consent withdrawal, please see our Privacy Policy.

Reviewer #1: No

Reviewer #2: No

**********

[NOTE: If reviewer comments were submitted as an attachment file, they will be attached to this email and accessible via the submission site. Please log into your account, locate the manuscript record, and check for the action link "View Attachments". If this link does not appear, there are no attachment files.]

To ensure your figures meet our technical requirements, please review our figure guidelines: https://journals.plos.org/plosone/s/figures

You may also use PLOS’s free figure tool, NAAS, to help you prepare publication quality figures: https://journals.plos.org/plosone/s/figures#loc-tools-for-figure-preparation.

NAAS will assess whether your figures meet our technical requirements by comparing each figure against our figure specifications.

# Author comment: Author response to Decision Letter 1

**DOI:** 10.1371/journal.pone.0351128.r002

7 May 2026

We thank the editor and reviewers for their valuable comments and suggestions, which have helped us to improve the quality of the manuscript. A detailed, point-by-point response to all reviewer and editor comments has been provided in the attached document ("Response to Reviewers").

For completeness, and as requested, we also reproduce our responses in the box below.

Reviewer #1:

We would like to thank the reviewer for the careful reading of our manuscript and for the constructive and valuable comments provided. We greatly appreciate the detailed feedback, which has helped us improve the clarity, precision, and overall quality of the manuscript.

In response to the reviewer’s suggestions, we have revised the manuscript to clarify key aspects of the sample description and competitive context, improve the reporting of variables and methodological procedures, and refine the wording in several sections to ensure greater accuracy and consistency. We have also expanded specific parts of the Introduction and Methods to better support the rationale and interpretation of the findings, and we have tempered certain statements in the Discussion and practical applications to better reflect the scope and limitations of the study. All changes have been carefully addressed and are detailed in the point-by-point responses below.

Abstract:

Fourteen elite female field

30 hockey players (eight of them international-level standard)

- what level was the rest of the athletes?

All players included in the study belonged to the same first team, which competes at the highest national and European level (Spanish national champions and European runners-up). The distinction made in the original version aimed to highlight that eight players were also members of the Spanish national team. However, we agree that this may have created ambiguity regarding the level of the remaining players. This has been clarified in the revised manuscript by revising the sample description in both the Abstract and Methods sections.

"During four high-level competitive matches "

what does this mean? What is the level of competition (national? intra squad? international?

We agree that the term “high-level competitive matches” was not sufficiently specific. The manuscript has been revised to clearly define the level of competition by specifying that the matches were official competitive fixtures played against top-level opponents (i.e., teams ranked in the top four of the national league or National Cup semi-finalists).

Intro:

Step frequency also

37 demonstrated very large associations with sprint, sprint·min⁻¹, HSR·min⁻¹, and maximal

38 acceleration

- please provide data to support "very large"

The correlation coefficients (r) and associated p-values have now been included in the revised manuscript to substantiate these statements.

The relationship

73 between these factors and performance has been reported in the case of SF, and it may be

74 affected differently by fatigue and competitive level [22,23].

- this sentence could use more information on the relationships between factors - is it inverse? positive? how is it affected by fatigue and comp level?

We agree that the original sentence lacked specificity regarding the nature of the relationships described. The text has been revised to clarify the direction of the relationship between step frequency (SF) and performance in the context of fatigue, as well as its modulation by competitive level.

GPS-derived physical performance metrics during elite

83 female field hockey matches.

- Which GPS metrics?

We have clarified the GPS-derived metrics by including representative examples (total distance, high-speed running, sprint distance, accelerations and decelerations, and maximum speed) in the Introduction, while avoiding excessive methodological detail.

Materials and methods:

96 Participants

97 Fourteen elite female hockey players (age 25.40 ± 4.48 years, height 1.68 ± 0.04 m and

98 body weight 58.53 ± 2.56 kg), including eight international-level players participated in

99 this study.

- What was the level of the remaining players?

We clarified the competitive level of the participants by specifying that all players belonged to the same first team, which were Spanish national champions and European runners-up. This contextual information reflects the elite level of all participants. Additionally, we specified that eight players were members of the Spanish national team.

Were they analyzed or grouped by position? this would be an important factor to include in game data. This is a major limitation.

Players were not grouped or analyzed according to playing position. This was due to the inclusion criterion requiring players to participate in all analyzed matches, which reduced the sample size from the original squad due to illness-related absences. In addition, some players performed different positional roles across matches, making positional classification inconsistent. For these reasons, and to avoid excessive model complexity given the number of variables analyzed, positional analysis was not performed. This aspect has now been acknowledged as a limitation in the revised manuscript.

During these matches, the average number of

114 goals scored by the studied team was 2.5 compared with 4.8 during the regular league

115 season. Goals conceded averaged 0.75 against top-four teams and 0.60 against lower 116 ranked teams.

- This feels random here. Maybe more context needed? Is this indicating the context of the games played?

We have clarified the context of the analyzed matches by specifying that they were played against top-ranked teams, representing higher competitive demands compared to regular league fixtures. To better support this, we included match outcome indicators (goals scored and conceded), which reflect the increased difficulty of these matches. Additionally, we clarified that all matches were performed within a three-week period following RSA testing, which can be considered sufficiently short to assume a relatively stable fitness status of the players.

Were all players played in every game? was just active time on pitch included in analysis?

We have clarified in the Participants section that only players who participated in all analysed matches were included in the study, which explains why the final sample did not include the entire squad. Additionally, we have specified in the GPS data section that relative variables were calculated based on the total time spent on the field during matches.

Table 1-4 - Horizontal lines between rows would improve readability

Horizontal lines between rows have been added to Tables 1–4 to improve readability, and we hope this facilitates clearer interpretation of the data.

276 DISCUSSION

277 Firstly, in line with the secondary aim, the use of averaged match data across four games with similar ranking teams proved to be a valid and reliable approach,

I think language coul dbe softened here, it does not necessarily prove antying, but is just 4 games so may suggest rather than prove

The language has been revised to adopt a more cautious tone, replacing definitive statements with more appropriate expressions

Practical applications like step frequency above 4hz and decrements below 4% feel like they are overstretching what the paper actually shows.

I think it is also important to acknowledge small sample size and use of correlational analyses in the limitaitons, and with any interpretations.

We agree that the proposed practical thresholds (e.g., step frequency > 4 Hz and performance decrements < 4%) should be interpreted with caution. These values were derived based on the mean step frequency observed in the present study and are supported by the magnitude of step frequency decrements reported in previous literature [22,23], where reductions of approximately 3–4% during RSA have been described. To address this concern, the text has been revised to present these values as tentative reference points rather than definitive thresholds.

Additionally, as suggested, we have explicitly acknowledged in the limitations section the relatively small sample size and the correlational nature of the analysis, emphasizing that the findings do not imply causality and should be interpreted with caution.

Reviewer #2:

Overall comments:

This manuscript examines the associations between repeated sprint ability (RSA) kinematic variables and locomotor activity in elite female field hockey players. The topic is relevant, and integrating RSA kinematics with GPS derived match metrics has the potential to contribute meaningfully to applied practice. The manuscript is generally well structured, and the rationale is clearly articulated. However, several important methodological and statistical details are missing, which limits confidence in the robustness of the findings.

The statistical analysis, in particular, requires greater methodological rigour. The manuscript does not justify the sample size or specify the variable on which the study was powered, and confidence intervals are not provided for key estimates. The reliance on simple correlations limits the ability to account for confounding factors or the repeated measures structure of the data. Mixed effects models or multivariable regression approaches could provide more robust insights by modelling within player variability and controlling for covariates. Addressing these issues would substantially strengthen the statistical validity and interpretability of the findings.

We would like to sincerely thank the reviewer for the thorough and insightful evaluation of our manuscript. We greatly appreciate the time and effort invested in providing such a detailed and constructive review. The comments have been extremely valuable in helping us improve the methodological clarity, statistical transparency, and overall interpretability of the study.

In response to these suggestions, we have carefully revised the manuscript, addressing all the points raised. Substantial improvements have been made, including clarification of methodological procedures, refinement of statistical reporting (e.g., inclusion of confidence intervals and assumptions checking), adjustments in terminology to ensure conceptual accuracy, and a more cautious interpretation of the findings in line with the correlational nature of the data. We have also incorporated additional references, expanded relevant sections of the Introduction and Methods, and explicitly acknowledged key limitations of the study. All changes are described in detail in the point-by-point responses provided below.

Title: Consider whether “match performance” is appropriate; the study examines locomotor activity, not performance outcomes.

We agree that the term “match performance” may be misleading, as the study specifically examines locomotor activity rather than direct performance outcomes. Accordingly, we have revised the title to better reflect the nature of the variables analyzed. We believe this change improves the accuracy and clarity of the manuscript.

Short title: Similarly, “match demands” is misleading. GPS/accelerometer data reflect activity profiles, not the true “demands” of match situations.

The short title has been revised to better reflect the nature of the variables analyzed.

Abstract: Replace “physical performance” with “GPS derived locomotor activity.”

Revised as suggested: ‘physical performance’ has been replaced with ‘GPS-derived locomotor activity’ in the Abstract.

L42: Fatigue development was not assessed; this claim should be removed.

The statement has been revised to remove any implication of fatigue assessment. The sentence now refers to the prediction of GPS-derived locomotor activity during competitive matches.

L49 – linking GPS to match outcomes is inappropriate. Greater locomotor activity may be a consequence of the standard of opponent and does not determine the ‘outcome’ (match result), as the sentence currently implies. See James et al. (2023). Relationships between opponent ranking and locomotor activity in international field hockey. International Journal of Sports Science & Coaching, 18(6), 2070-2081.

Feels like first paragraph is mostly redundant, given the number of preceding hockey GPS papers. I think the authors could probably start at Paragraph 2.

We agree that linking locomotor activity directly to match outcomes may be inappropriate, as match results are influenced by multiple contextual factors. Accordingly, the sentence has been revised to remove any implication of a direct relationship with match outcomes. In addition, the introductory paragraph has been streamlined to reduce redundancy and improve clarity.

L62 – 66. Suggest to attach the specific citation to each of these points about opponent / playing style / match importance. Environmental conditions are another modifying factor here, for example see James et al. (2022). Increased air temperature decreases high-speed, but not total distance, in international field hockey. Temperature, 9(4), 357–372.

We have revised the sentence to associate each contextual factor with specific references and have incorporated environmental conditions, including air temperature, as an additional factor influencing locomotor activity, as recommended.

L71 – 73 – the importance of kinematic factors is not explained. Authors may wish to expand on this. Why is it important to understand? Does it guide training prescription?

We thank the reviewer for this comment. We agree that the importance of kinematic factors was not sufficiently explained. The text has been revised to clarify their relevance by highlighting that sprint velocity is determined by the interaction between step length and step frequency (V = SL × SF), and that their analysis provides additional insight into the mechanisms of performance and fatigue, which may help to inform training prescription.

L78 – Clarify what is meant by “improving the ecological validity of RSA testing.” Are you implying RSA protocols should be modified based on these correlations?

The term ‘ecological validity’ has been replaced to better reflect the practical relevance of RSA testing in relation to GPS-derived locomotor activity.

L83 – ‘As a preliminary step’ not needed

The wording has been revised to remove ‘preliminary step’ and clarify the role of this analysis in supporting the use of averaged match data. For this reason, the results of the secondary aim are presented first in the Results section, as they provide the basis for the subsequent correlational analyses between match averages and RSA performance.

L85 – why were four matches selected?

The four matches were selected to ensure consistency with the RSA testing context and to include matches against top-ranked opponents, thereby reducing variability related to competitive level. This selection criterion is now clarified in the Methods section.

L85 – do you mean absolute total meters or intensity? (per min?). This is important to distinguish because you cannot control the playing time of the players.

As indicated in the Methods section, both absolute values and relative values normalized to individual playing time were included in the analysis.

L90 – what is similarly ranked?

The term ‘similarly ranked’ has been clarified. The analyzed team consistently ranked first or second in the national league over the past 10 years, and comparisons were made against teams occupying top positions in the league standings or reaching the semi-finals of the National Cup. This information has been clarified in the Methods section.

L91 – stability is inappropriate term for describing variability

The term ‘stability’ has been revised to ‘low match-to-match variability’ to more accurately describe the consistency of the measured variables.

L92 – this is not competitive performance, but locomotor activity.

The term ‘competitive performance’ has been replaced with ‘locomotor activity’ throughout the manuscript to ensure terminological accuracy.

L98 – Consider using the McKay et al. performance calibre framework to contextualise your players. doi:0.1123/ijspp.2021-0451

The performance calibre framework proposed by McKay et al. has been incorporated into the manuscript. Players were classified a

Attachment Submitted filename: Response to Reviewer 2.docx

# Peer review: Decision Letter 1

**DOI:** 10.1371/journal.pone.0351128.r003

**Authors:** Paul Goods

18 May 2026

<div>PONE-D-26-08660R1

Sprint velocity and step frequency during repeated sprint ability testing are associated with high-intensity locomotor activity in elite female field hockey

PLOS One

Dear Dr. González-Frutos,

Thank you for submitting your manuscript to PLOS ONE. After careful consideration, we feel that it has merit but does not fully meet PLOS ONE’s publication criteria as it currently stands. Therefore, we invite you to submit a revised version of the manuscript that addresses the points raised during the review process.

Please submit your revised manuscript by Jul 02 2026 11:59PM. If you will need more time than this to complete your revisions, please reply to this message or contact the journal office at plosone@plos.org. When you're ready to submit your revision, log on to https://www.editorialmanager.com/pone/ and select the 'Submissions Needing Revision' folder to locate your manuscript file.

Please include the following items when submitting your revised manuscript:

- A letter that responds to each point raised by the academic editor and reviewer(s). You should upload this letter as a separate file labeled 'Response to Reviewers'.
- A marked-up copy of your manuscript that highlights changes made to the original version. You should upload this as a separate file labeled 'Revised Manuscript with Track Changes'.
- An unmarked version of your revised paper without tracked changes. You should upload this as a separate file labeled 'Manuscript'.

If you would like to make changes to your financial disclosure, please include your updated statement in your cover letter. Guidelines for resubmitting your figure files are available below the reviewer comments at the end of this letter.

If applicable, we recommend that you deposit your laboratory protocols in protocols.io to enhance the reproducibility of your results. Protocols.io assigns your protocol its own identifier (DOI) so that it can be cited independently in the future. For instructions see: https://journals.plos.org/plosone/s/submission-guidelines#loc-laboratory-protocols. Additionally, PLOS ONE offers an option for publishing peer-reviewed Lab Protocol articles, which describe protocols hosted on protocols.io. Read more information on sharing protocols at https://plos.org/protocols?utm_medium=editorial-email&utm_source=authorletters&utm_campaign=protocols.

As the corresponding author, your ORCID iD is verified in the submission system and will appear in the published article. PLOS supports the use of ORCID, and we encourage all coauthors to register for an ORCID iD and use it as well. Please encourage your coauthors to verify their ORCID iD within the submission system before final acceptance, as unverified ORCID iDs will not appear in the published article. *Only* the individual author can complete the verification step; PLOS staff *cannot* verify ORCID iDs on behalf of authors.

We look forward to receiving your revised manuscript.

Kind regards,

Paul SR Goods

Academic Editor

PLOS One

Journal Requirements:

If the reviewer comments include a recommendation to cite specific previously published works, please review and evaluate these publications to determine whether they are relevant and should be cited. There is no requirement to cite these works unless the editor has indicated otherwise.

Please review your reference list to ensure that it is complete and correct. If you have cited papers that have been retracted, please include the rationale for doing so in the manuscript text, or remove these references and replace them with relevant current references. Any changes to the reference list should be mentioned in the rebuttal letter that accompanies your revised manuscript. If you need to cite a retracted article, indicate the article’s retracted status in the References list and also include a citation and full reference for the retraction notice.

Additional Editor Comments (if provided):

Congratulations on the substantial edits to the manuscript which have been positively received by both reviewers.

You will note both reviewers have a remaining minor query which should be responded to in a final round of revisions.

[Note: HTML markup is below. Please do not edit.]

Reviewers' comments:

Reviewer's Responses to Questions

**Comments to the Author**

1. If the authors have adequately addressed your comments raised in a previous round of review and you feel that this manuscript is now acceptable for publication, you may indicate that here to bypass the “Comments to the Author” section, enter your conflict of interest statement in the “Confidential to Editor” section, and submit your "Accept" recommendation.

Reviewer #1: All comments have been addressed

Reviewer #2: (No Response)

**********

2. Is the manuscript technically sound, and do the data support the conclusions?

The manuscript must describe a technically sound piece of scientific research with data that supports the conclusions. Experiments must have been conducted rigorously, with appropriate controls, replication, and sample sizes. The conclusions must be drawn appropriately based on the data presented.

Reviewer #1: Yes

Reviewer #2: Yes

**********

3. Has the statistical analysis been performed appropriately and rigorously?

Reviewer #1: No

Reviewer #2: Yes

**********

4. Have the authors made all data underlying the findings in their manuscript fully available?

The PLOS Data policy requires authors to make all data underlying the findings described in their manuscript fully available without restriction, with rare exception (please refer to the Data Availability Statement in the manuscript PDF file). The data should be provided as part of the manuscript or its supporting information, or deposited to a public repository. For example, in addition to summary statistics, the data points behind means, medians and variance measures should be available. If there are restrictions on publicly sharing data—e.g. participant privacy or use of data from a third party—those must be specified.

Reviewer #1: Yes

Reviewer #2: No

**********

5. Is the manuscript presented in an intelligible fashion and written in standard English?

PLOS ONE does not copyedit accepted manuscripts, so the language in submitted articles must be clear, correct, and unambiguous. Any typographical or grammatical errors should be corrected at revision, so please note any specific errors here.

Reviewer #1: Yes

Reviewer #2: Yes

**********

6. Review Comments to the Author

Please use the space provided to explain your answers to the questions above. You may also include additional comments for the author, including concerns about dual publication, research ethics, or publication ethics. (Please upload your review as an attachment if it exceeds 20,000 characters)

Reviewer #1: Thanks to the authors for the detailed revisions and responses to the reviewer comments. The manuscript has improved in clarity, contextual detail, and interpretation of the findings.

I appreciate the more cautious framing of the correlational findings and practical applications, along with the added acknowledgment of limitations related to the sample size, positional analysis, and study design.

I still think the small sample size and correlational/statistical approach should be considered when interpreting the findings and practical implications, and I think the overall contribution of the paper remains relatively modest. However, I think the authors have addressed the major concerns appropriately within the scope of the dataset, and I have no further major concerns.

Reviewer #2: Well done on making considerable changes. I think the manuscript is much better positioned bow, especially as many limitations are transparently reported.

The only minor comment i would make to the authors is to encourage them to again reconsider the use of 'relative distance' - just because other research has published this, is not a rationale to use scientifically incorrect terminology. Distance is not being normalized to some factor, it is simply divided by time, which provides speed.

**********

7. PLOS authors have the option to publish the peer review history of their article (what does this mean?). If published, this will include your full peer review and any attached files.

If you choose “no”, your identity will remain anonymous but your review may still be made public.

**Do you want your identity to be public for this peer review?** For information about this choice, including consent withdrawal, please see our Privacy Policy.

Reviewer #1: No

Reviewer #2: No

**********

[NOTE: If reviewer comments were submitted as an attachment file, they will be attached to this email and accessible via the submission site. Please log into your account, locate the manuscript record, and check for the action link "View Attachments". If this link does not appear, there are no attachment files.]

To ensure your figures meet our technical requirements, please review our figure guidelines: https://journals.plos.org/plosone/s/figures

You may also use PLOS’s free figure tool, NAAS, to help you prepare publication quality figures: https://journals.plos.org/plosone/s/figures#loc-tools-for-figure-preparation.

NAAS will assess whether your figures meet our technical requirements by comparing each figure against our figure specifications.

# Author comment: Author response to Decision Letter 2

**DOI:** 10.1371/journal.pone.0351128.r004

21 May 2026

Reviewer #1:

Thanks to the authors for the detailed revisions and responses to the reviewer comments. The manuscript has improved in clarity, contextual detail, and interpretation of the findings.

I appreciate the more cautious framing of the correlational findings and practical applications, along with the added acknowledgment of limitations related to the sample size, positional analysis, and study design.

I still think the small sample size and correlational/statistical approach should be considered when interpreting the findings and practical implications, and I think the overall contribution of the paper remains relatively modest. However, I think the authors have addressed the major concerns appropriately within the scope of the dataset, and I have no further major concerns.

We sincerely thank the reviewer for the positive evaluation of the revised manuscript and for acknowledging the substantial improvements in clarity, contextualization, interpretation, and reporting of limitations. We appreciate the reviewer’s constructive feedback throughout the review process, which has contributed significantly to improving the quality of the manuscript.

Reviewer #2:

Well done on making considerable changes. I think the manuscript is much better positioned bow, especially as many limitations are transparently reported.

The only minor comment i would make to the authors is to encourage them to again reconsider the use of 'relative distance' - just because other research has published this, is not a rationale to use scientifically incorrect terminology. Distance is not being normalized to some factor, it is simply divided by time, which provides speed.

Thank you for this insightful comment and for the positive evaluation of the revised manuscript. We agree that the term “relative distance” may be conceptually imprecise, as the variable reflects distance covered per unit of time and therefore represents an average speed measure rather than a normalized distance metric. Accordingly, the terminology has been revised throughout the manuscript by replacing “total distance per minute” and its abbreviation “TD·min⁻¹” with “average speed” and “Avg speed”, respectively, to improve conceptual and scientific accuracy.

Attachment Submitted filename: Response_to_Reviewer_2_auresp_2.docx

# Editor report: Decision Letter 2

**DOI:** 10.1371/journal.pone.0351128.r005

**Authors:** Paul Goods

24 May 2026

Sprint velocity and step frequency during repeated sprint ability testing are associated with high-intensity locomotor activity in elite female field hockey

PONE-D-26-08660R2

Dear Dr. González-Frutos,

We’re pleased to inform you that your manuscript has been judged scientifically suitable for publication and will be formally accepted for publication once it meets all outstanding technical requirements.

Within one week, you’ll receive an e-mail detailing the required amendments. When these have been addressed, you’ll receive a formal acceptance letter and your manuscript will be scheduled for publication.

An invoice will be generated when your article is formally accepted. Please note, if your institution has a publishing partnership with PLOS and your article meets the relevant criteria, all or part of your publication costs will be covered. Please make sure your user information is up-to-date by logging into Editorial Manager at Editorial Manager® and clicking the ‘Update My Information' link at the top of the page. For questions related to billing, please contact billing support.

If your institution or institutions have a press office, please notify them about your upcoming paper to help maximize its impact. If they’ll be preparing press materials, please inform our press team as soon as possible -- no later than 48 hours after receiving the formal acceptance. Your manuscript will remain under strict press embargo until 2 pm Eastern Time on the date of publication. For more information, please contact onepress@plos.org.

Kind regards,

Paul SR Goods

Academic Editor

PLOS One

# Editor report: Acceptance letter

**DOI:** 10.1371/journal.pone.0351128.r006

**Authors:** Paul Goods

PONE-D-26-08660R2

PLOS One

Dear Dr. González-Frutos,

I'm pleased to inform you that your manuscript has been deemed suitable for publication in PLOS One. Congratulations! Your manuscript is now being handed over to our production team.

At this stage, our production department will prepare your paper for publication. This includes ensuring the following:

* All references, tables, and figures are properly cited

* All relevant supporting information is included in the manuscript submission,

* There are no issues that prevent the paper from being properly typeset

You will receive further instructions from the production team, including instructions on how to review your proof when it is ready. Please keep in mind that we are working through a large volume of accepted articles, so please give us a few days to review your paper and let you know the next and final steps.

Lastly, if your institution or institutions have a press office, please let them know about your upcoming paper now to help maximize its impact. If they'll be preparing press materials, please inform our press team within the next 48 hours. Your manuscript will remain under strict press embargo until 2 pm Eastern Time on the date of publication. For more information, please contact onepress@plos.org.

You will receive an invoice from PLOS for your publication fee after your manuscript has reached the completed accept phase. If you receive an email requesting payment before acceptance or for any other service, this may be a phishing scheme. Learn how to identify phishing emails and protect your accounts at https://explore.plos.org/phishing.

If we can help with anything else, please email us at customercare@plos.org.

Thank you for submitting your work to PLOS ONE and supporting open access.

Kind regards,

PLOS ONE Editorial Office Staff

on behalf of

Dr. Paul SR Goods

Academic Editor

PLOS One
